# Optimization of glass scrap recovery and reuse in road construction for promising physicochemical stabilization

**DOI:** 10.1038/s41598-024-62862-x

**Published:** 2024-06-05

**Authors:** Noureddine Ouslimane, Jaouad Bensalah, Hanane Barebita, Mohamed Ebntouhami, Abdel-Rhman Z. Gaafar, Hiba-Allah Nafidi, Youssouf Ali Younous, Mohammed Bourhia, Mustapha Belfaquir

**Affiliations:** 1 Advanced Materials and Process Engineering Laboratory, Chemistry Department, Faculty of Sciences, Ibn Tofail University, BP 133, Kenitra, Morocco; 2 Control Laboratory, Building and Public Works Tests and Studies, TEST-BUILDING, Kenitra, Morocco; 3 Laboratory of Advanced Materials and Process Engineering (LMAGP), Faculty of Sciences, University Ibn Tofail, BP 133, Kenitra, Morocco; 4 Organic Chemistry, Catalysis and Environment Laboratory, Chemistry Department, Faculty of Sciences, Ibn Tofail University, BP 133, Kenitra, Morocco; 5 Botany and Microbiology Department, College of Science, King Saud University, P.O. Box 2455, 11451 Riyadh, Saudi Arabia; 6 Department of Food Science, Faculty of Agricultural and Food Sciences, Laval University, 2325 Rue de l’Université, Quebec City, QC G1V 0A6 Canada; 7 Evangelical College, BP 1200, N’Djamena, Chad; 8 Department of Chemistry and Biochemistry, Faculty of Medicine and Pharmacy, Ibn Zohr University, 70000 Laayoune, Morocco

**Keywords:** Proctor, Swelling, CBR, Characterization, Glass, Clay raw, Infrastructure, Chemistry, Materials science

## Abstract

Waste glass is hugely present in Morocco, and can be recycled for many geotechnical purposes, including road construction. In contrast, earthworks often produce significant amounts of clay waste that lack the necessary technical criteria for use as barriers. The present work aimed to study the influence of the addition of glass waste on the evolution of the mechanical characteristics of clays stabilized with crushed glass (particles less than 63 μm). The work consists of carrying out CBR, Proctor, and shear tests on natural clay taken as a reference and mixtures (clay-crushed glass) at different percentages. Results showed that the addition of glass to clay decreases the swelling and compaction indices along with modifying the intrinsic characteristics of the clay.

## Introduction

Clays are considered problem soils in the construction of road infrastructure. They are generally associated with high compressibility, high plasticity, and evolutionary behavior. This paper aims to investigate the mechanical properties of fine soils, namely clay sourced from the Ouezzane area when subjected to compaction and treatment with glass waste. To conduct this study, experiments were performed for various durations of cure. The concept of sustainable development, which has garnered global attention, is undeniably connected to the development of civil engineering structures, including road infrastructure^1,2^.

Clay soils have long been recognized for their sensitivity to hydric variations during seasonal cycles consequently, posing significant challenges in construction projects both in Morocco and worldwide. Notably, this swelling phenomenon is inherent to clay soils and is influenced by the mineralogical, chemical, and mechanical properties of the constituents^3^. Concurrently, the growing ubiquity of glass waste in landfills has emerged as a significant environmental concern. The accumulation of glass waste, arising from its widespread usage, has motivated innovative approaches to its sustainable management. Glass, known for its amorphous structure and abundant silicon content, presents opportunities for repurposing in various applications^4–7^. The differentiation between several categories of glass trash, such as container glass waste (e.g., bottles) and flat glass waste (e.g., windows), is of particular significance. Every category has distinct difficulties and possibilities for recycling, which is a crucial aspect of modern waste management endeavors. Despite its inherent fragility, glass has exceptional durability, surviving in landfills for thousands of years. Research efforts have been driven by the need to effectively handle and recycle glass trash. These efforts focus on integrating glass waste into building materials, which not only addresses waste issues but also improves material qualities^8–10^. Glass is known for its notable characteristics such as high compressive strength, minimal water absorption, and resistance to chemical degradation. These traits make it an attractive option for enhancing the mechanical properties of many materials, including clay soils^11^. The present research aligns seamlessly with this broader context, centering on the valorization of industrial glass debris and household glass waste as aggregates for road pavements, particularly in foundation layers^12–14^. Although prior studies have explored various applications of clay in civil engineering, including road construction, the specific synergy of clay and glass in road construction materials remains relatively unexplored. This study's objective is to bridge this knowledge gap by delving into the mechanical behavior of mixtures comprising clay and glass, thereby offering valuable insights into their viability for road engineering.

The research methodology involves the meticulous preparation of clay and glass mixtures in varying proportions, followed by a comprehensive assessment of their mechanical properties. Standard laboratory-based mechanical tests commonly employed in road engineering, including Proctor compaction tests, California Bearing Ratio (CBR) tests, and shear tests, are conducted on the reconstituted samples. These tests provide invaluable data concerning the performance and suitability of clay-glass mixtures for application in road construction.

Glass incorporation in Portland cement, including Glass scrap, is used in the production of Portland cement. Glass that has been recovered is used as the primary component for clinker, an additive for cement, or even as a component for concrete. Glass is now being tested with Portland cement. Before being sent through a glass grinder, these glass pieces undergo a washing process. The outcome of the grinding process yields a powder that successfully traverses the 600 µm sieve. The X-ray analysis reveals that the substance in question is an amorphous powder. The behavior of glass fragments in Portland cement was investigated in a recent work^16^. Based on the findings of these investigations, it can be inferred that the use of glass residue, when reduced to small particles, in conjunction with cement, has potential. Portland serves as a binding agent. Numerous experimental investigations have been conducted to use glass residue as aggregate in bituminous coatings. The coating in question is often referred to as Glasphalt. From 1969 to 1988, over 45 regions in the United States and Canada effectively used Glasphalt on various locations, including local roads, parking lots, and high-speed roadways (highways). The Connecticut Department of Transportation (Conn DOT) carried out a technical implementation study as well as an economic analysis of the use of glass scraps in bituminous coatings. This study made it possible to identify certain behaviors and particularities linked to the use of glass in bituminous coatings, namely:—Lack of adhesion between glass aggregates and bitumen, low density, broken pieces of glass, and tire damage. Thus, the Glasphalt used as a base coat provided a better result since we no longer encountered the problem of tire damage;—The size of the glass grains should be limited to 10 mm;—Under ideal conditions, Glasphalt costs approximately 15% more than conventional bituminous mixture.

Several studies aimed to use glass debris in the construction of roadways and incorporate a percentage of glass residue into conventional roadway aggregates, while others aimed to use 100% broken glass in certain parts of the roadway structure. The literature showed that the use of scrap glass as construction materials did not cause any particular problem, both for the producer and for the contractor^15^. Thus, the scrap glass mixes perfectly with the aggregates, which requires less water to obtain an acceptable density. Construction equipment and vehicles were not damaged by the use of broken glass as a base layer construction material. The comparison of construction costs^16^ between “mixed” materials based on glass residue and those composed of natural materials demonstrated that “mixed” materials cost relatively more than natural materials. Part of these costs were attributed to the additional treatment conditions (sorting and crushing) of the glass and the process of mixing glass and natural aggregates.

A previous study^17^ demonstrated that the use of broken glass in the coating did not deteriorate its properties, as long as its content did not exceed 15%, but it seems that the true optimum is 7.5%. As part of this project, the authors also studied the influence on the bearing capacity (CBR) of the use of a percentage of glass in a glass-silt mixture while specifying that the CBR values are not modified as long as the percentage of glass does not exceed 15%. The freezing susceptibility of broken glass was studied in an earlier study^18^, on broken glass, containing approximately 1% passing through an 80-micron sieve, demonstrated its susceptibility to freezing ranging from negligible to very low. In some cases, the glass is mixed with the aggregate during crushing, while in other cases^19^ it is mixed in the field by mixing with a grader by forming windrows after spreading glass on aggregate^19^. Noteworthy, experiments carried out in a previous study^20^ involved the use of 300 tonnes of crushed glass in a road embankment as the glass was first placed to a thickness of 200 to 250 mm and then compacted using a tractor followed by a compactor.

Earlier works^21^ were conducted on the physical behavior of glass debris. Two glass samples were used in this project. The first sample consisted of debris from domestic waste (food, juice, beer, soft drinks, and occasional pieces of china) from a quarry in Pennsylvania, while the second sample included industrial glass waste (automobiles, television screens, and electronic devices) purchased from a merchant in the city whose activity consisted of washing and storing scrap glass and reselling it to the pharmaceutical and glazier industries in the region. The sample from the trader was less well sorted and contained more non-glass material than the sample from the quarry. Almost 3.4% of waste was found from labels, metal and plastic lids. This sample gave off a very strong smell of mold. The first sample contained little odor and only 0.8% of waste other than broken glass.

It is essential to acknowledge that glass fragments are increasingly prevalent in landfills, serving diverse purposes ranging from packaging and decorations to construction materials^22–29^. Notably, the integration of glass waste into civil engineering applications has recently gained significant attention, with a focus on its potential use in addressing challenges posed by swelling clays and enhancing public infrastructure^30–37^.

Building upon this established body of research, the current study aims to further elucidate the mechanical behavior of clay and glass mixtures, particularly their potential contribution to road construction^31,32^. Upon the identification of the selected materials, the mixtures, comprised of clay and glass, undergo a battery of main mechanical tests commonly used in road engineering. These tests include modified Proctor compaction tests, California Bearing Ratio (CBR) tests conducted both before and after immersion, as well as various road tests.

The subsequent outcomes derived from various sample compositions are then evaluated about the criteria set by relevant standards, therefore offering significant perspectives on the viability of using clay-glass mixes in the context of road building. This research attempt signifies a notable advancement in tackling the environmental issues linked to glass waste and the technical complexities presented by clay soils. This work makes a valuable contribution to the existing body of knowledge on sustainable road-building techniques by examining the mechanical interaction between clay and glass. It also presents a promising opportunity for enhancing the discipline of civil engineering in line with global sustainability goals.

## Methods

### Setting study

**Figure 1:**
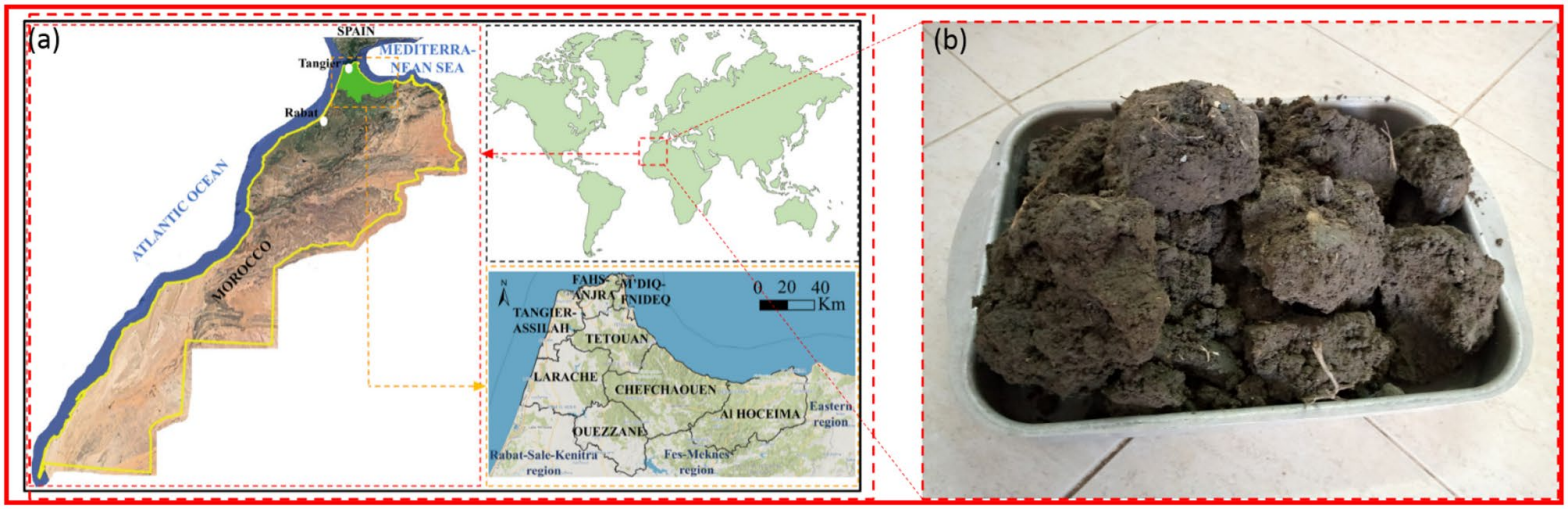
(**a**) Map of the study area at Ouazzane Province, Morocco**—**https://www.google.com/maps). (**b**) photograph of samples.

The characterization of the study area holds significant importance within the framework of our project as it allows for the identification of areas where the materials requiring treatment are present^38–40^. The aforementioned phase is of utmost importance in the execution of a thorough geological and geotechnical categorization of the various materials found within the designated research region. By using this classification procedure, it is possible to accurately ascertain the optimal soils that are appropriate for the different treatment approaches^41–45^. The collection of samples was conducted at a depth of roughly 1.0 m. Following the extraction process, the soil was carefully enclosed in plastic bags and then brought to the laboratory to conduct geotechnical identification and characterization tests. In the present geotechnical investigation, our primary objective is to examine the expansion and strengthening of the RP 4527 road segment, which extends from KP 8 + 300 to KP 19 + 000, located in the Ouazzane Province of Morocco (Fig. 1).

### Classification of water forms and their stability in clay matrices

The impact of water on the rheological and mechanical characteristics of clays, such as plasticity, compaction, and cohesion, is of significant importance in defining their behavior. Water may exist in many forms inside the clay matrix, displaying differing levels of stability. The various forms may be categorized according to their degrees of stability, which span from the highest stability to the lowest stability^18^.

#### The water of constitution

Constitutional water, also known as coupled water, is a kind of water that is chemically bonded to clay molecules. It is an essential component of the material's chemical makeup. The elimination of combined water is achieved only by the application of elevated temperatures to the material, resulting in the disruption of chemical bonds that connect the water molecules^18,46^.

#### Hydration water

This particular sort of water is often known as zeolite water, bound water, or adsorbed water. It is characterized by its physical attachment to the surface of solid materials, mostly occurring on the walls of capillaries. Through electrical processes, it creates a thin layer on the surface of particles and makes up about 6 to 7% of the overall water content^18^.

#### The absorbed water

Hygroscopic water refers to the water that accumulates within the outer layers surrounding the clay sheets. The heat of adsorption for this water is approximately 2650 kJ/kg^18^.

#### Open water

In moist soils, including those with similar characteristics, water circulates within the macrospores that are present between soil aggregates. This movement allows water to freely flow within the medium, however, complete evaporation occurs when a soil sample is subjected to an oven at a constant temperature of 105 °C^3,33^.

### Stabilization techniques

Various stabilizing strategies are regularly used among the widely accessible alternatives^19^. Nevertheless, the choice of a particular technique is impacted by other aspects such as financial considerations, soil properties, the length of the process, the availability of supplies, and environmental circumstances. The present investigation used the mechanical stabilization methodology, namely the modified Proctor process and the applicable clay techniques^19^.

#### Mechanical stabilization

##### Compaction

The compaction of clay soil is a crucial step in stabilization, as it aims to reduce the soil's porosity and achieve optimal characteristics including the moisture content (W_OPM_) and the maximum dry density (ρd_OPM_). The compaction parameters are determined through the modified Proctor test^19^.

##### Drainage

The use of drainage systems is a well embraced and conventional method for effectively managing surplus water in diverse environments. This methodology involves the use of several techniques, such as trench drainage, cardboard drains, and vertical glass drains^19^.

##### Substitution

When the coating of undesired material is very thick, it may be difficult or impracticable to remove it. In instances of this kind, an alternate methodology is the excavation of the soil to a certain depth, followed by the substitution of such soil with appropriate materials such as glass or gravel. However, it is important to acknowledge that this method incurs significant expenses^41–44^.

### Methods and techniques

#### Sample preparation

The mechanical tests were performed on samples prepared as mixtures with different proportions of natural clay and glass. The samples were designated as follows:

- Sample 1: Mixture (natural clay + 0% glass waste).
- Sample 2: Mixture (natural clay + 10% glass waste).
- Sample 3: Mixture (natural clay + 20% glass waste).

#### Tests performed

The prepared samples were subjected to various mechanical tests in the laboratory, which included:

- Modified Proctor compaction tests: These tests assess the compaction suitability of the mixtures and determine their mechanical characteristics at the W_OPM_ and ρd_OPM_^45–47^.
- Bearing capacity tests before and after immersion: These tests evaluate the bearing capacity of the mixtures under different conditions. The immediate bearing capacity index (IPI) is determined to assess the load-bearing capacity of the mixtures under traffic loads generated by construction machinery while the CBR index after immersion (CBRimm) is determined after the samples are subjected to adverse hygrometric conditions (CBR index after immersion: CBR_imm_)^48^.
- Shear tests: The shear tests involved applying a constant load to a soil sample and measuring its mechanical characteristics through linear shearing.
- These tests involve subjecting a constant load to a soil sample and measuring its mechanical characteristics through linear shearing. The objective was to establish the intrinsic shear behavior of the clay and glass mixtures and determine important parameters such as cohesion (c) and angle of internal friction (φ)^49^.

### Test method

#### Modified proctor tests

**Figure 2:**
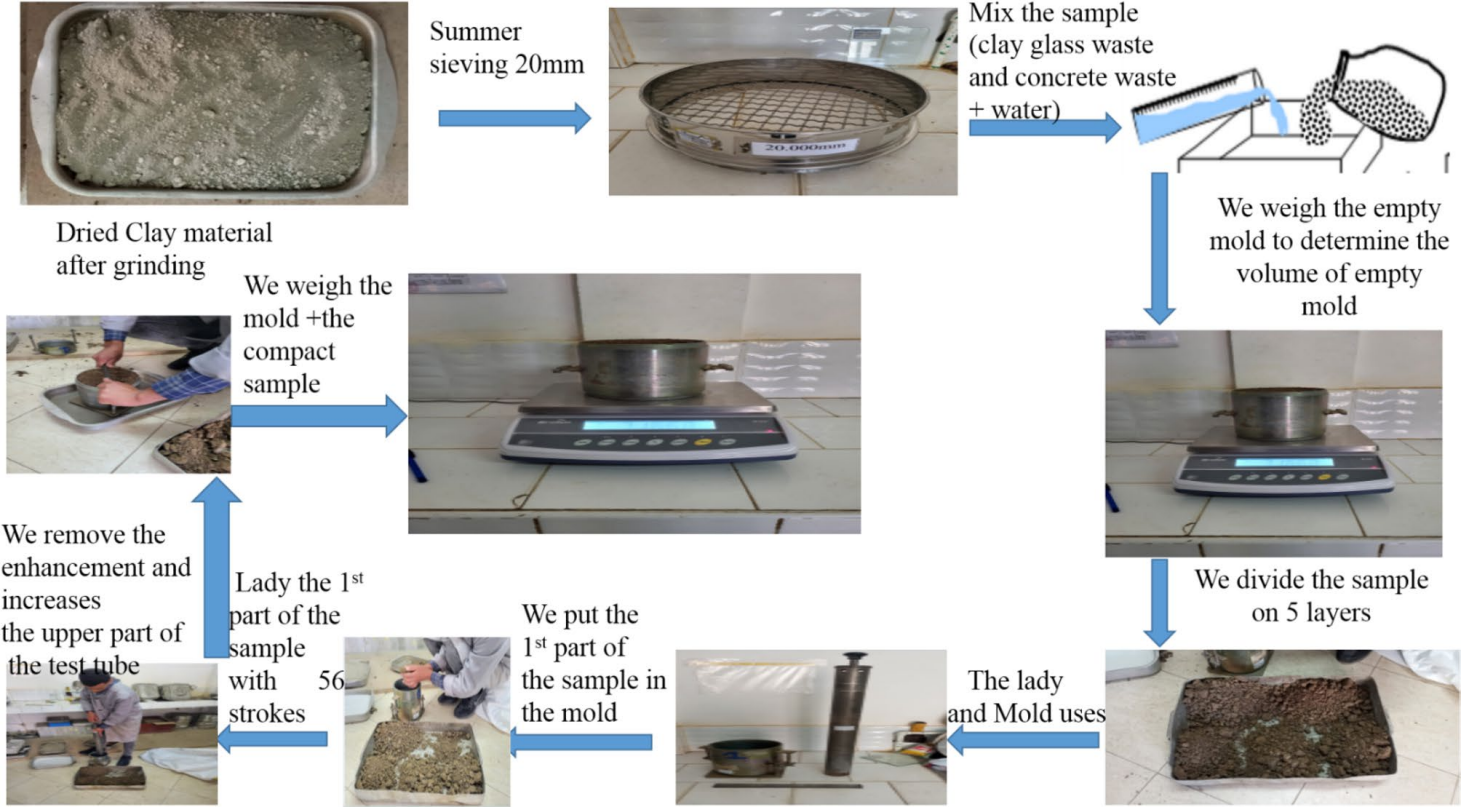
Diagram illustrating the modified Proctor method.

In the present study, the used waste glass constituted damaged car windshields, household waste such as waste linked to commercial activities, construction waste, and waste from public services. Importantly, samples were first characterized by observing the appearance of the solution and the presence or absence of suspended matter in the water-clay-glass mixture and subjected to the proctor tests as illustrated in Fig. 2.

#### Direct shear tests on the box

**Figure 3:**
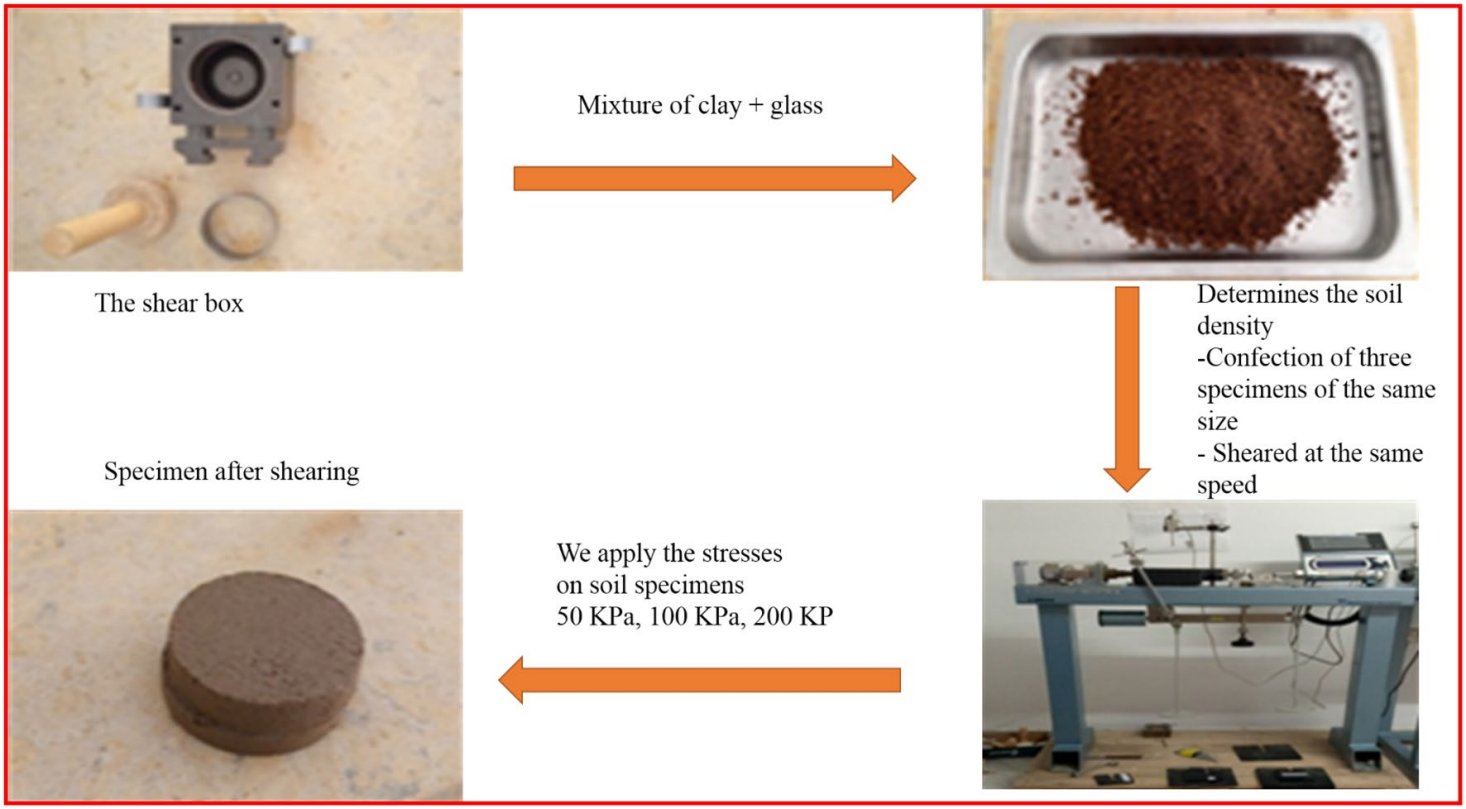
Diagram illustrating the direct box shear method.

In this analysis, we aimed to ascertain the soil density resulting from the mixture of clay with glass by subjecting the different samples to shear tests as depicted in Fig. 3.

## Results and discussions

All the different tests described in this study were conducted in the Clay Mechanics Soil Laboratory, located in the Ouazzane province. Tests were conducted in triplicate assays and results are presented as means ± stand deviations.

### Particle size analysis by sieve analysis of exploited clay

**Figure 4:**
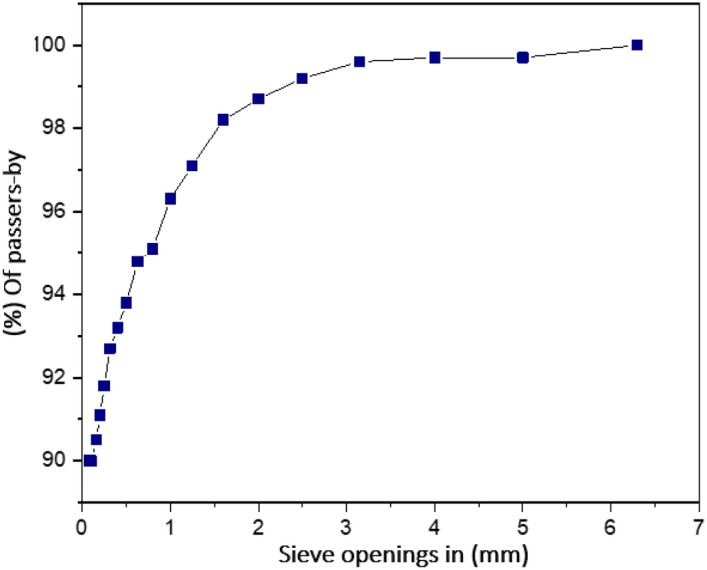
Grain size curve of the clay that was studied.

Clay soil is often characterized as a loose or malleable substance consisting of particles with dimensions less than 80 µm and 2 µm in size^43^. The particles in question consist of a diverse range of constituents, which also include clay minerals and non-clay minerals including quartz, feldspars, carbonates, and degraded organic materials^44^. The clay soil's geotechnical capabilities, hydraulic behavior, and mechanical features are primarily determined by the composition and ratio of clay minerals within the soil matrix. The clay mineral composition has a considerable impact on the geotechnical qualities of clay soil, such as plasticity, compressibility, and swelling^3^. The grain size curve of the clay that was reevaluated in this study is shown in Fig. 4.

Grain size analysis shows that there is less clay in the soil sampled than in other samples. The categorization of fine soils will provide further validation for these results. Soils in Ouazzane could have more compressibility, as the sample shows a finer particle size distribution, according to the curve.

### Physicochemical characterization of clay materials

The physical characteristics of the clay materials under study are presented in Table 1.

**Table 1:** The main characteristics present in the tested soils.

Physical characteristics		Unity	Results	Chemical characteristics	Unity	Results
Weight water content (w)		(%)	29.5 ± 0.5	VBS methylene blue value	(g/100 g)	8 ± 0.2
Granulometric	Dmax	–	–			
	< 2	mm	99 ± 1.0			
	< 0.08	mm	90 ± 2.0			
Liquidity limit (W_L_)		(%)	75 ± 3.0			
Plastic limit (W_P_)		(%)	31 ± 1.5			
Plasticity index (IP)		(%)	44 ± 2.5			
Consistency index (Ic)		(%)	1.03 ± 0.03			
Classification (GMTR)					A3	

The plasticity index (IP) of the clay samples exceeds 40 indicating high plasticity, while the consistency index (Ic) exceeds 1 indicating a firm soil behavior. These results classify the studied material as highly plastic and firm according to important standards. Furthermore, the material falls within the threshold range that distinguishes clay soils from highly clayey soils as indicated by the value of Verbeek's plasticity index (VBS) which is 8. This classification confirms the clayey nature of the material.

The organic content of the clay samples is found to be low, suggesting minimal presence of organic matter in the soil. Additionally, the material is characterized as moderately aggressive, belonging to type A3 based on the classification system employed.

### Physico-chemical characterization of glass waste

Glass waste is characterized by a number of important physicochemical qualities, such as its chemical composition, density, hardness, transparency, optical properties, melting point, thermal expansion, electrical insulation, color, environmental effect, and fracture properties. Furthermore, Tables 2 and 3 provide information on the physical qualities of waste glass based on their hardness and density values.

**Table 2:** Main physicochemical characteristics of glass waste.

Features	Designation	Unit	Results
Calcium oxide	CaO	(%)	8.60
Potassium oxide	K_2_O	(%)	0.055
Sodium oxide	Na_2_O	(%)	13.63
Iron oxide	Fe_2_O_3_	(%)	0.77
Sulfur trioxide	SO_3_	(%)	0.19
Titanium dioxide	TiO_2_	(%)	0.06
Aluminium oxide	Al_2_O_3_	(%)	0.55
Magnesium oxide	MgO	(%)	4.070
Zirconium dioxide	ZrO_2_	Ppm	67.00
Manganese oxide	MnO	Ppm	255.00
Barium oxide	BaO	Ppm	23.00
Carbon monoxide	CO	Ppm	< 3
Chromium oxide	Cr_2_O_3_	Ppm	19.00
Oxyde de vanadium	V_2_O_5_	Ppm	< LLD
Chlorides	CL	Ppm	144.00
Chrome	Cr	Ppm	13.00
Lead	Pb	Ppm	< LLD
Cadmium	Cd	Ppm	< LLD
Mercure	Hg	Ppm	< LLD

**Table 3:** Hardness and density values.

Product	Hardness	Density (g/cm^3^)
FGN5	4.8–5	2.502

### Particle size analysis by use of sieve analysis of glass

**Figure 5:**
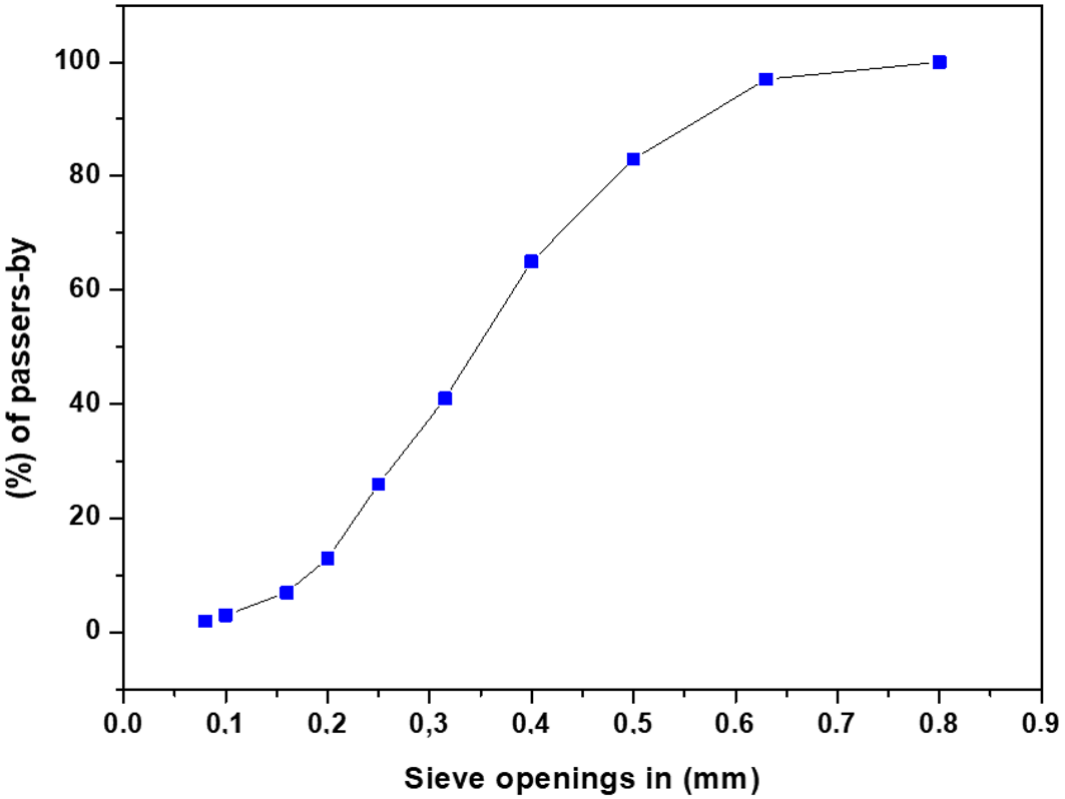
Glass particle size curve used.

In the present research work, household waste such as waste linked to commercial activities, construction waste, and waste from public services (school, administration, etc.) were used analyzed by use of sieve Analysis of glass. The glass was washed with water to remove organic matter (paper and glue) after drying. The particle size analysis of crushed glass results in the granular class 0/1 presented in Fig. 5.

### X-ray diffraction (XRD)

**Figure 6:**
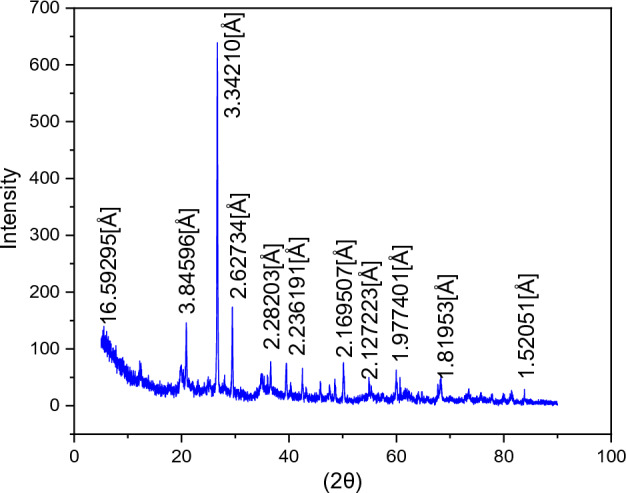
X-ray diffraction spectra of the Clay raw (K: kaolinite, Q: quartz, I:illite).

The study of the structural and crystalline properties, as well as the phases present in the deposited thin layers, necessitates the use of the X-ray diffraction technique. X-ray diffraction (XRD) is a fundamental method to investigate crystalline materials. One of the most significant applications of X-ray diffractometry is the identification of phases present in a sample and the determination of their structures. X-ray diffraction enables the study of materials composed of many crystals with arbitrary orientations (Fig. 6). These crystals exhibit a series of parallel and equidistant planes known as lattice planes (hkl). When a monochromatic and parallel X-ray beam illuminates the sample, the rays are diffracted in a specific direction by each family of lattice planes whenever the Bragg condition is satisfied.

The method is based on the experimental application of Bragg's law.

$$ {\text{2d}}\;{\text{sin}}\theta \, = \,{\text{n}}\lambda $$

Where

n: The order of the diffraction.

λ: The wavelength of the emitting source.

d_(hkl)_: The spacing between two successive parallel planes of the crystal lattice.

θ: The angle of diffraction.

XRD analysis, as represented in Fig. 6, was conducted to investigate the clay sample. The XRD patterns of the clay material are shown in the results. The diffraction peaks of the clay material are observed at 2θ = 3.84°, 3.24°, and 16.59°, corresponding to d-spacings of 2.62 Å, 2.28 Å, and 1.52 Å, respectively. The clay raw material consists of a mixture of quartz, illite, and kaolinite^18,19^.

It seems you have provided information regarding the composition and characterization of a clay sample using different analytical techniques such as X-ray Fluorescence (XRF) and X-ray Diffraction (XRD). Here is a potential interpretation and elaboration of this information:

The clay specimen was subjected to X-ray Fluorescence (XRF) examination, resulting in the acquisition of information pertaining to its elemental makeup. Table 4 displays the findings of this investigation. The clay composition mostly consists of silica (SiO2), calcium (Ca), iron (Fe), alumina (Al2O3), and magnesium oxides (MgO). The aforementioned elements are often encountered constituents of clays and have a pivotal influence on their physicochemical characteristics.

**Table 4:** The composition of the clay sample was further characterized by use of X-ray analysis.

Pos. [2θ]	Height [cts]	FWHM left[2θ]	d-Spacing [Å]	Rel. int. [%]
5.324433	25.655560	0.944640	16.59795	06.59
18.080420	45.590670	0.118080	4.90644	11.71
20.878220	63.776390	0.078720	4.25484	16.38
23.126960	35.562420	0.157440	3.84596	09.13
26.673560	389.404000	0.137760	3.34210	100.00
29.457130	355.796100	0.157440	3.03232	91.37
31.001630	18.284700	0.118080	2.88468	04.70
34.126720	43.340810	0.157440	2.62734	11.13
36.047280	39.572850	0.157440	2.49164	10.16
36.615650	37.786020	0.196800	2.45426	09.70
39.489370	80.902270	0.236160	2.28203	20.78
40.333600	12.139470	0.236160	2.23619	03.12
41.230980	15.724740	0.236160	2.18957	04.04
42.495100	22.499280	0.118080	2.12732	05.78
43.228940	48.236570	0.236160	2.09289	12.39
45.893280	07.994876	0.472320	1.97740	02.05
47.106290	26.544080	0.236160	1.92927	06.82
47.587550	51.944370	0.236160	1.91088	13.34
48.601490	57.742120	0.196800	1.87336	14.83
50.136920	39.753800	0.196800	1.81953	10.21
55.062650	07.225497	0.629760	1.66785	01.86
57.536630	16.508710	0.314880	1.60188	04.24
59.994380	25.915850	0.236160	1.54201	06.66
60.863650	13.139960	0.314880	1.52205	03.37
64.748470	10.223080	0.314880	1.43980	02.63
68.284930	22.568490	0.314880	1.37360	05.80
73.241970	05.891373	0.944640	1.29239	01.51
75.715930	05.045967	0.472320	1.25620	01.30
79.931690	10.758470	0.314880	1.20022	02.76
81.539680	08.144985	0.787200	1.18057	02.09
83.912570	09.851171	0.314880	1.15312	02.53

These findings align with those obtained from X-ray Diffraction (XRD) analysis, which confirmed the presence of quartz as the main source of silica (SiO2). Quartz is an abundant mineral composed of silicon dioxide (SiO2), and its presence is consistent with the XRF results.

Furthermore, the observation of higher content of calcium oxide (CaO) and magnesium oxide (MgO) denotes that the predominant exchangeable cations in the clay sample are calcium ions (Ca2 +) and magnesium ions (Mg2 +). Exchangeable cations refer to positive ions present in the clay's structure that can be swapped with other ions in the environment. The CaO and MgO content thus indicates the abundance of these cations in the sample, which can have significant implications for ion exchange and water retention properties of the clay.

Both X-ray Fluorescence (XRF) analysis and X-ray Diffraction (XRD) analysis provide comprehensive information on the composition and structure of the clay sample under investigation. The comprehensive characterization of the clay is of utmost importance in comprehending its prospective qualities and uses, including industrial, environmental, and scientific domains.

### Infrared (IR)

**Figure 7:**
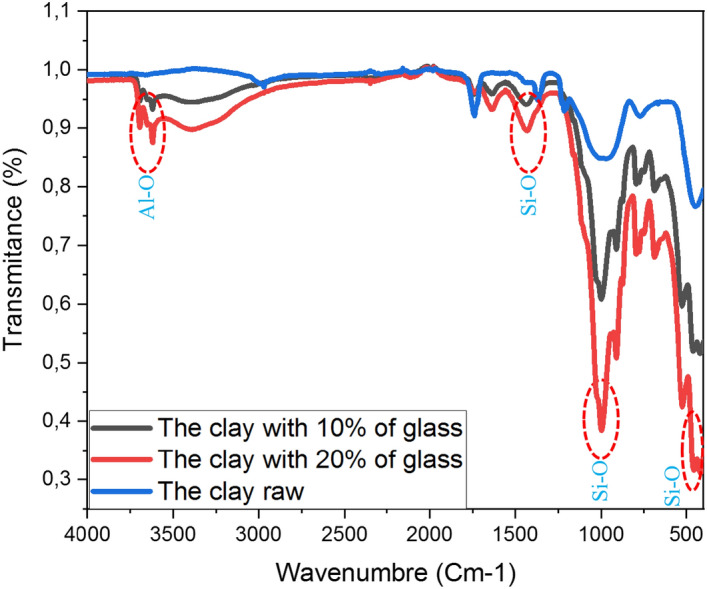
Infrared spectra of clay raw, Clay with 10% glass, and Clay with 20% glass.

FT-IR analysis was conducted to verify the presence of different functional groups in the extracted clay sample. The obtained FT-IR spectrum is demonstrated in Fig. 7. The spectrum shows a distinct and broad peak observed at 3390 cm^-1^, indicating the stretching vibration of the O–H group. This peak confirms the presence of the O–H functional group in the clay sample.

The literature references^8,50^ and the results of the FT-IR analysis suggest that the main chemical components found in the clay sample share structural similarities with histamine. These components have multiple bonds and heteroatoms (Oxygen atoms), which make them capable of interacting with the surface structure of metals through a donor–acceptor mechanism. This interaction results in the creation of a protective layer at the interface between the carbon clay and glass.

The swelling phenomenon of glass upon adsorption involves the participation of various functional groups, such as oxide, amino, and hydroxyl groups. In this study, the clay sample's cell surface contains Si–O and Al-O groups, which are indicated by the peaks observed at specific wavenumbers (1030 cm^-1^, 1066 cm^-1^, 1434 cm^-1^, and 3536 cm^-1^) in the provided figure (Fig. 7). These functional groups likely contribute to the interaction between the clay and the glass surface.

The clay sample seems to have seen an improvement in its swelling capacity as a result of the treatment procedure, especially when coupled with different proportions of glass. This observation implies the participation of the MgO (magnesium oxide) moiety in the process of swelling. The provided information suggests that the observed swelling behavior is influenced by the interaction among the clay, glass, and maybe the MgO group. The present book examines the results of the structural similarity between the chemical constituents found in the clay sample and histamine, their interaction with the glass surface, the existence of distinct functional groups on the surface of the clay cells, and the involvement of the MgO group in the process of swelling. The present research aims to investigate the role of various components and their interactions in the development of a protective layer and the enhancement of the clay sample's swelling capacity when exposed to glass.

### EDX and SEM analysis of adsorbent

**Figure 8:**
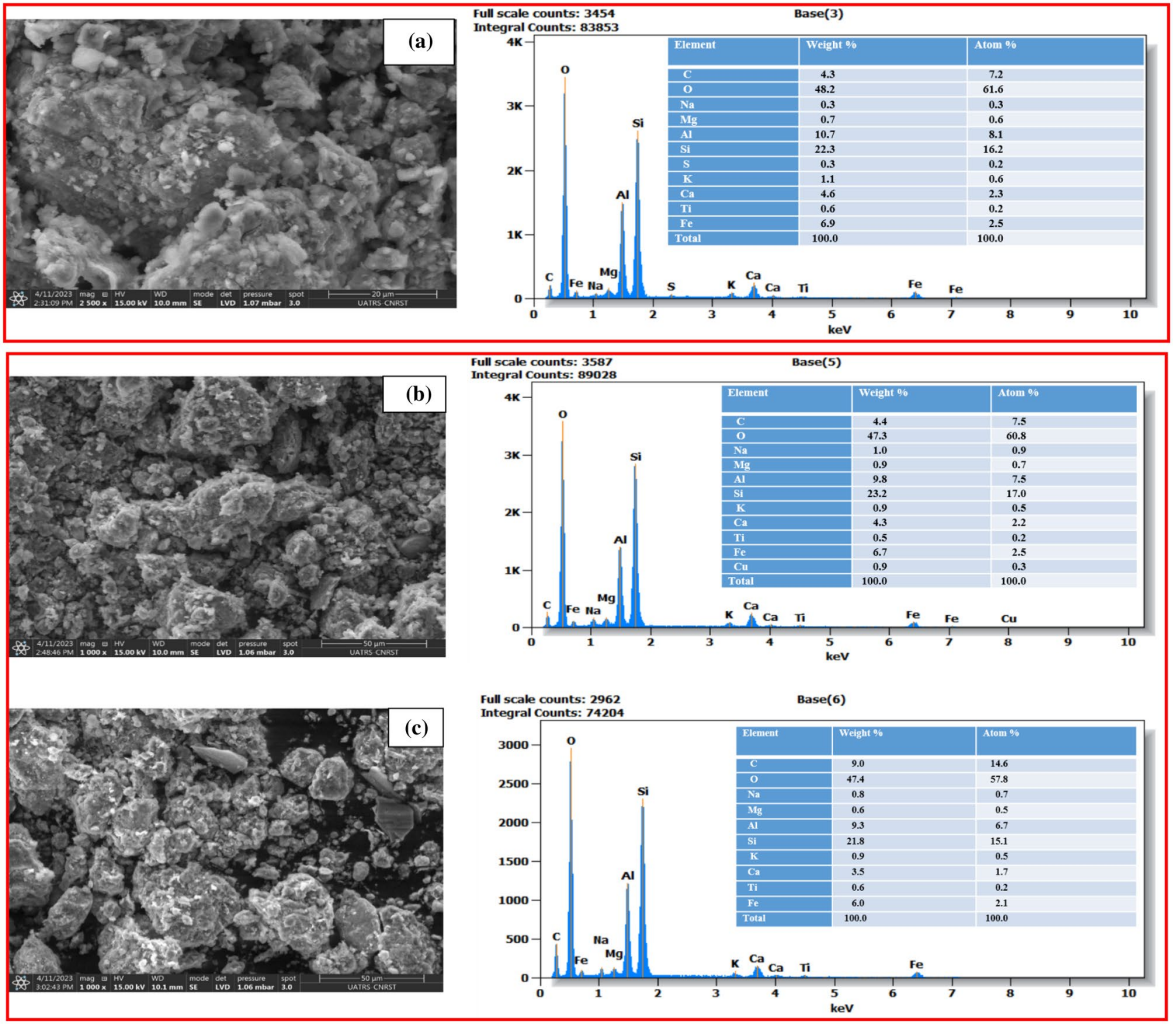
SEM and EDX of the Clay raw (**a**), Clay with 10% glass (**b**), and Clay with 20% glass (**c**).

Various SEM images were obtained for the adsorbent clay raw material, including images before and after the addition of the swelling agent, as well as after the swelling treatment (Fig. 8). The SEM images of the clay raw material (Fig. 8a) show the presence of porosity on the surface, indicating the presence of microcavities and irregular molecules. This porous morphology is advantageous for the adsorption technique. However, when the clay raw material is treated with the swelling agent at different percentages, significant white clouds appear (Fig. 8b) due to the interaction with the swelling agent.

The EDX diffraction diagram (Fig. 8) offers valuable insights into the impact of the integration of glass into the clay raw material via the technological procedure. It emphasizes the development of a more noticeable uneven porosity after this integration. The observed augmentation in porosity seems to have a significant impact on the facilitation of metal ion migration from the glass into the clay substrate. This phenomenon is seen across various ratios of glass incorporation.

More specifically, the heightened porosity is advantageous as it generates more interaction sites between the glass and the clay material. This irregular porosity provides spaces and pathways for the diffusion of metal ions present in the glass. These metal ions can migrate through these porous spaces and come into contact with compounds present in the clay material.

The study findings also indicated the presence of various elemental compounds in the clay raw material after chemical treatment, whereby varied ratios of crystalline glass have been used. Elements such as silicon, aluminum, calcium, and other elements found in both glass and clay are among the elemental compounds that may be present. The ultimate composition of the adsorbent clay material may be influenced by the change in quantities of crystalline glass, which in turn affects its ability to adsorb and retain metal ions.

The EDX diffraction diagram and observations regarding the elemental compounds provide strong evidence that the incorporation of glass into the clay material leads to increased porosity and improved ionic interactions, while also influencing the chemical composition of the clay material. These findings are crucial for understanding the underlying mechanisms of this technique and for its potential application in various fields, such as water purification or waste treatment.

By use of advanced characterization methods like energy-dispersive X-ray spectroscopy (EDX) and scanning electron microscopy (SEM), it is possible to observe the distribution of elements and the structure of the changed clay samples. The aforementioned data provide empirical evidence supporting the dispersion of glass waste particles inside the clay matrix, therefore bolstering the concept of physical interactions between the phases. There are both potential and constraints associated with the integration of recovered glass trash into expanded clay for road building. Although the mechanical qualities may experience some deterioration when glass debris is added, the major benefits of this strategy include recyclability and environmental sustainability.

### Modified proctor tests

**Figure 9:**
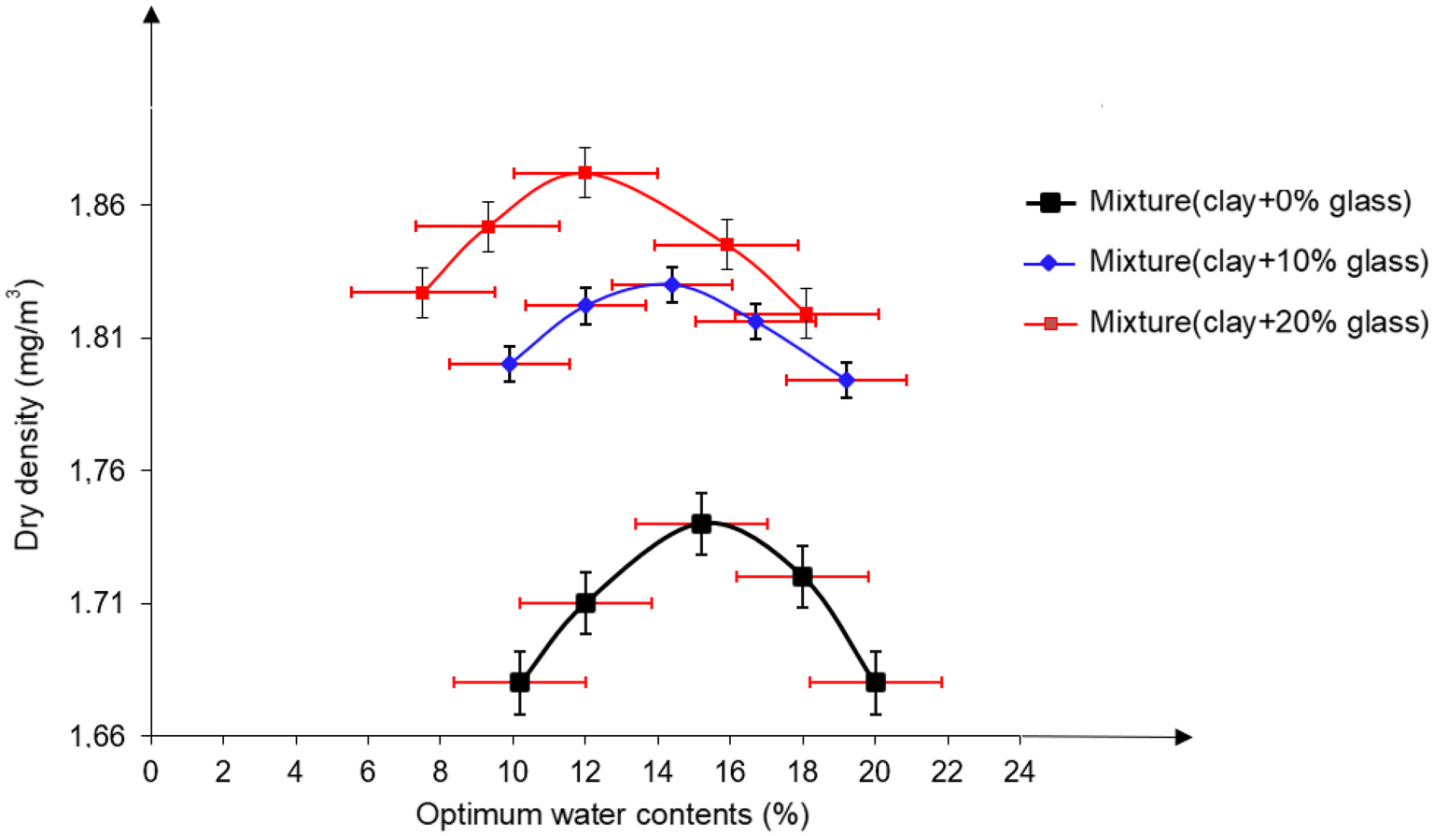
Proctor curves for studied mixtures.

**Figure 10:**
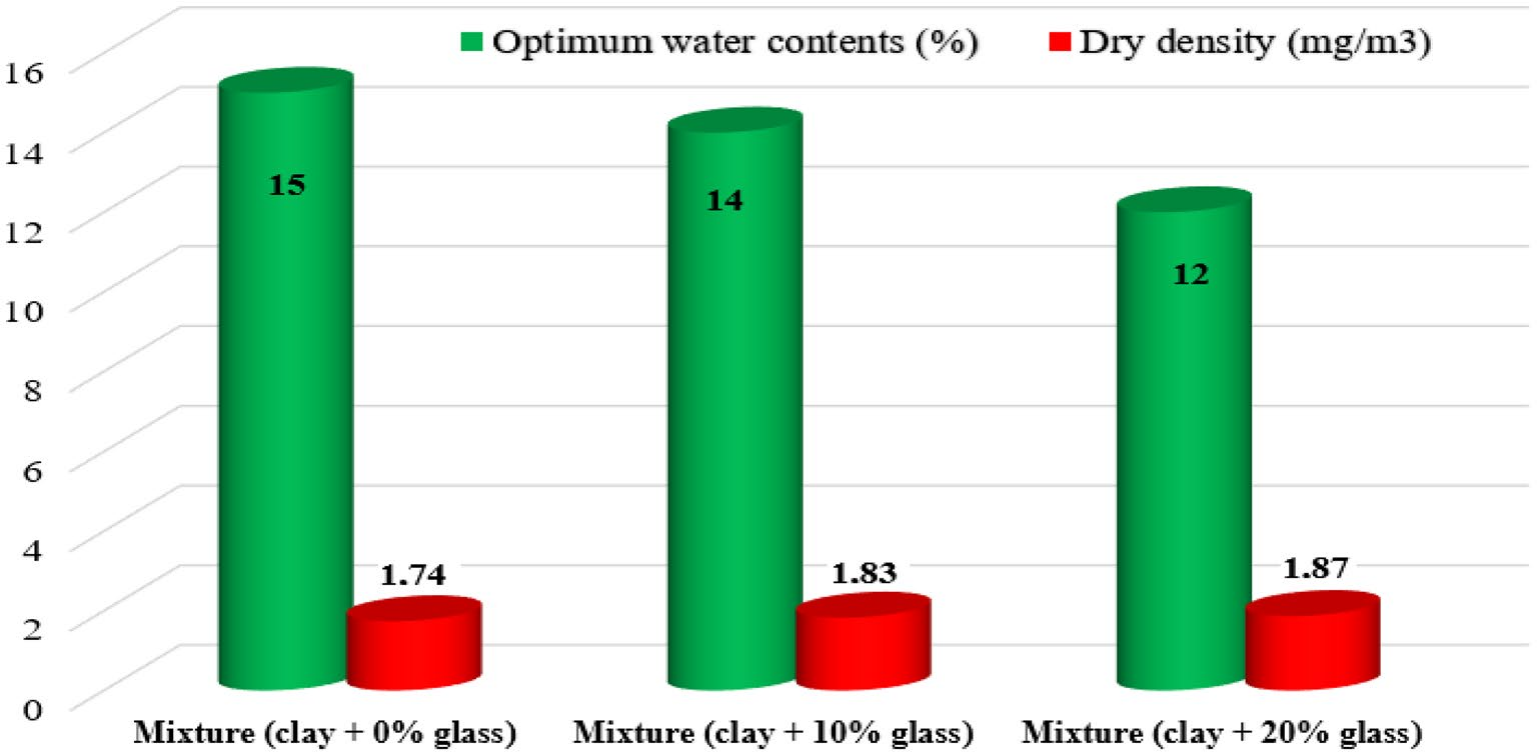
Evolution of compaction parameters as a function of glass content.

The compaction tests were done using the modified Proctor method reported elsewhere^53^. The Proctor curves, depicted in Figs. 9 and 10 illustrate the impact of glass addition on the compaction behavior of the mixtures. It is evident that the presence of glass progressively reduces the sensitivity of the mixtures to water particularly after the initial 10% glass addition. The Proctor curves for the mixtures containing glass exhibit a less rounded shape compared to the clay alone. This effect becomes more pronounced with higher glass contents. The reduced sensitivity of the mixtures with glass to water can be attributed to the glass occupying a significant proportion of the mixtures, thereby limiting their water sensitivity.

The present study concerns the mechanical stabilization technique (modified proctor) applied to Ouazzane clay (Table 5). Soil compaction is a very important step in stabilization to reduce soil porosity based on optimal compaction characteristics (γdopm and Wopt), which are determined by the normal or modified Proctor test.

**Table 5:** Proctor results of clay soil before and after treatment.

	Dry density (mg/m3)	Optimum water contents (%)
Mixture (clay + 0%glass)	1.74 ± 0.001	15 ± 0.01
Mixture (clay + 10%glass)	1.83 ± 0.001	14 ± 0.01
Mixture (clay + 20%glass)	1.87 ± 0.001	12 ± 0.01

Stabilization by adding glass fragments is the most widespread soil treatment technique that enables:

- Avoid large-scale earthworks when replacing poor soils.
- Clayey soils are rapidly given a good consistency at dosages of between 0 and 20%. This also demonstrates the economic benefits of this process.
- Modification of soil properties.

The choice of glass debris is made based on laboratory tests, site tests, and cost price. The figure shows that the Proctor curve for treated soil is displaced to the left and upwards about the curve for natural soil. This shift is all the more pronounced when the soil reacts well with waste glass.

Treatment with glass shards therefore reduces water content and increases the maximum value of dry bulk density that can be achieved. Numerous studies have shown that stabilizing clay soils with glass shards transforms them into firm soils, improving their strength and permeability and stabilizing their volume after swelling and shrinkage. These results enable us to qualify the mixes studied from a compaction point of view as acceptable and interesting materials for use in medium to heavy-traffic pavements.

It should be noted that the results could vary according to the origin of the waste glass used. Notably, the glass waste dimension and its density could affect the results, e.g. after grinding, the density and dimension of glass increase, which probably affects intrinsic parameters such as cohesion, friction angle, dry density, and Proctor Optimum, which in turn can increase its CBR index.

### CBR bearing capacity tests before and after immersion

**Figure 11:**
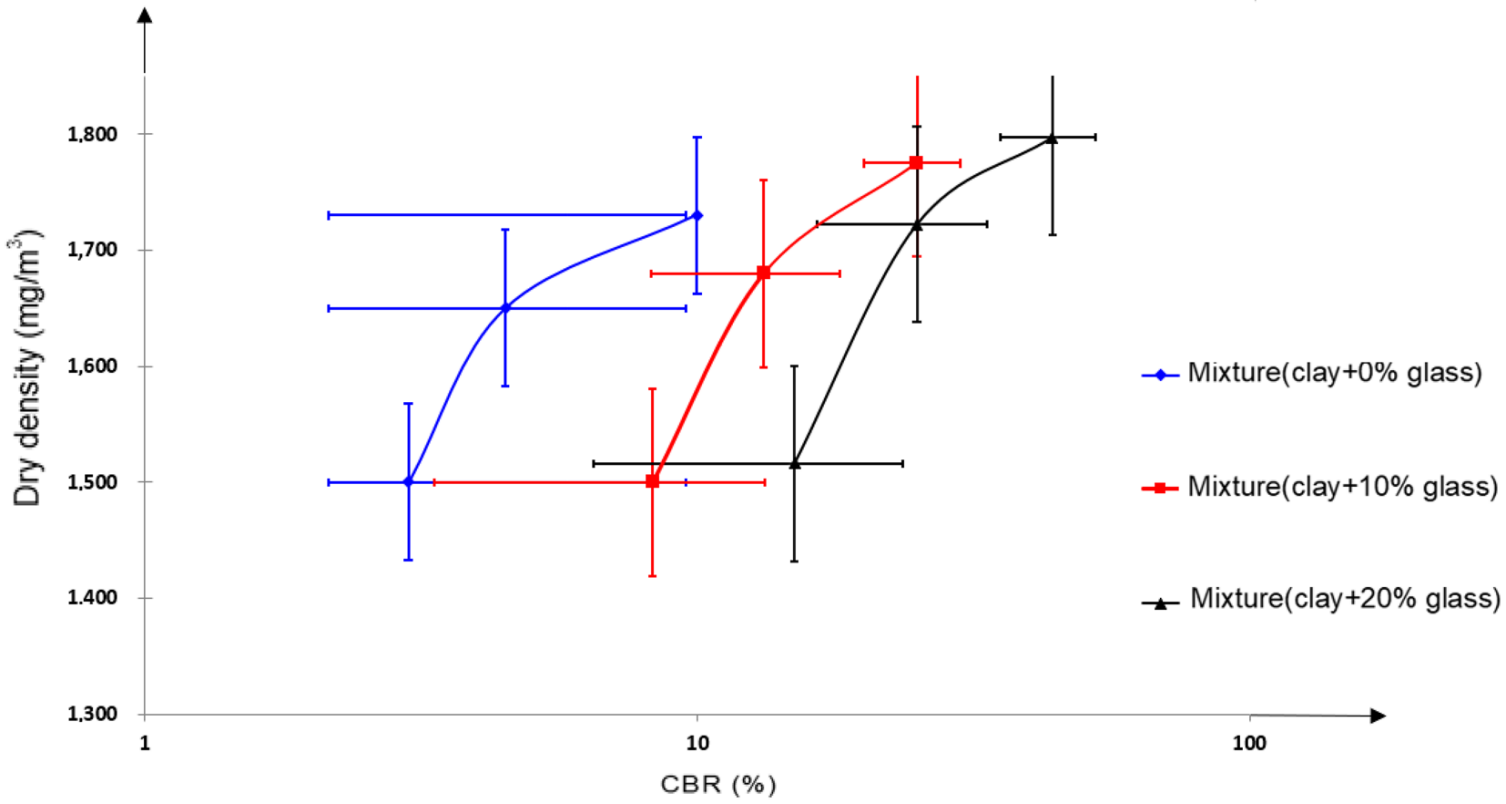
CBR test results after mix immersion.

**Figure 12:**
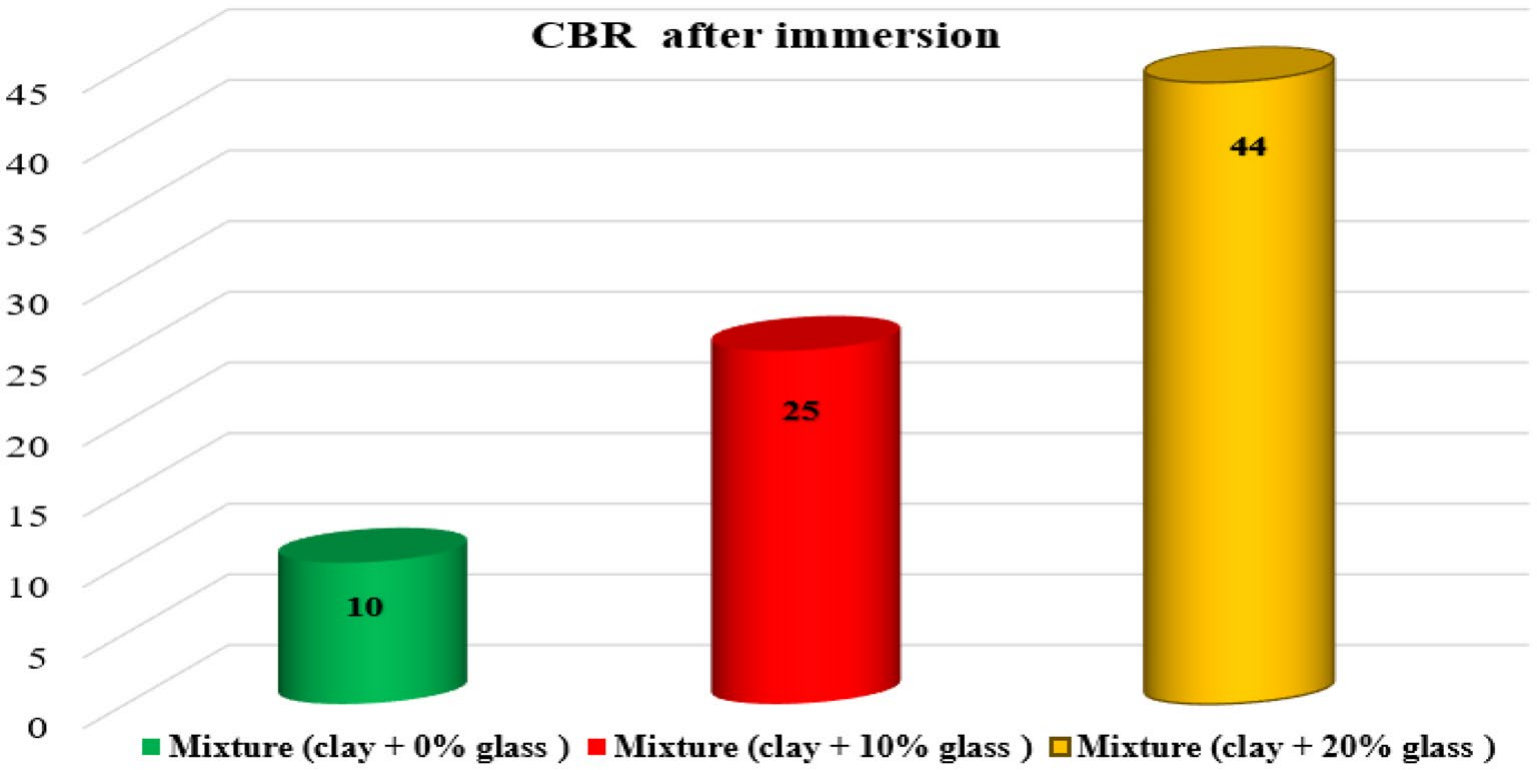
CBR test results after immersion.

The reconstituted samples of clay and glass fragments were moistened to their optimal water content for compaction replicating their actual state during implementation. Subsequently, CBR tests are conducted on these samples before and after immersion, following the guidelines specified in the standard^54^. These tests enable the assessment of their bearing capacity after immersion, commonly referred to as CBRimm, and the results are presented in Figs. 11, 12 and Table 6

**Table 6:** CBR results after immersion of clay soil before and after treatment.

	Dry density (mg/m3)	CBR (%)
Mixture (clay + 0%glass)	1.65 ± 0.001	4.5 ± 0.2
Mixture (clay + 10%glass)	1.68 ± 0.001	13.2 ± 0.7
Mixture (clay + 20%glass)	1.72 ± 0.001	25.0 ± 1.3

The submerged CBR index values obtained after treatment comply with the recommendations of the French GTR earthworks guide. The results also show that in all cases, the immersed CBR index of the treated clay specimens increases proportionally with glass content and curing time. Referring to the lift classes, we can say that the materials studied develop very interesting lifts. The samples studied are respectively lift (class S4) and very high-lift (class S5) materials.

#### Direct shear tests at the box

**Figure 13:**
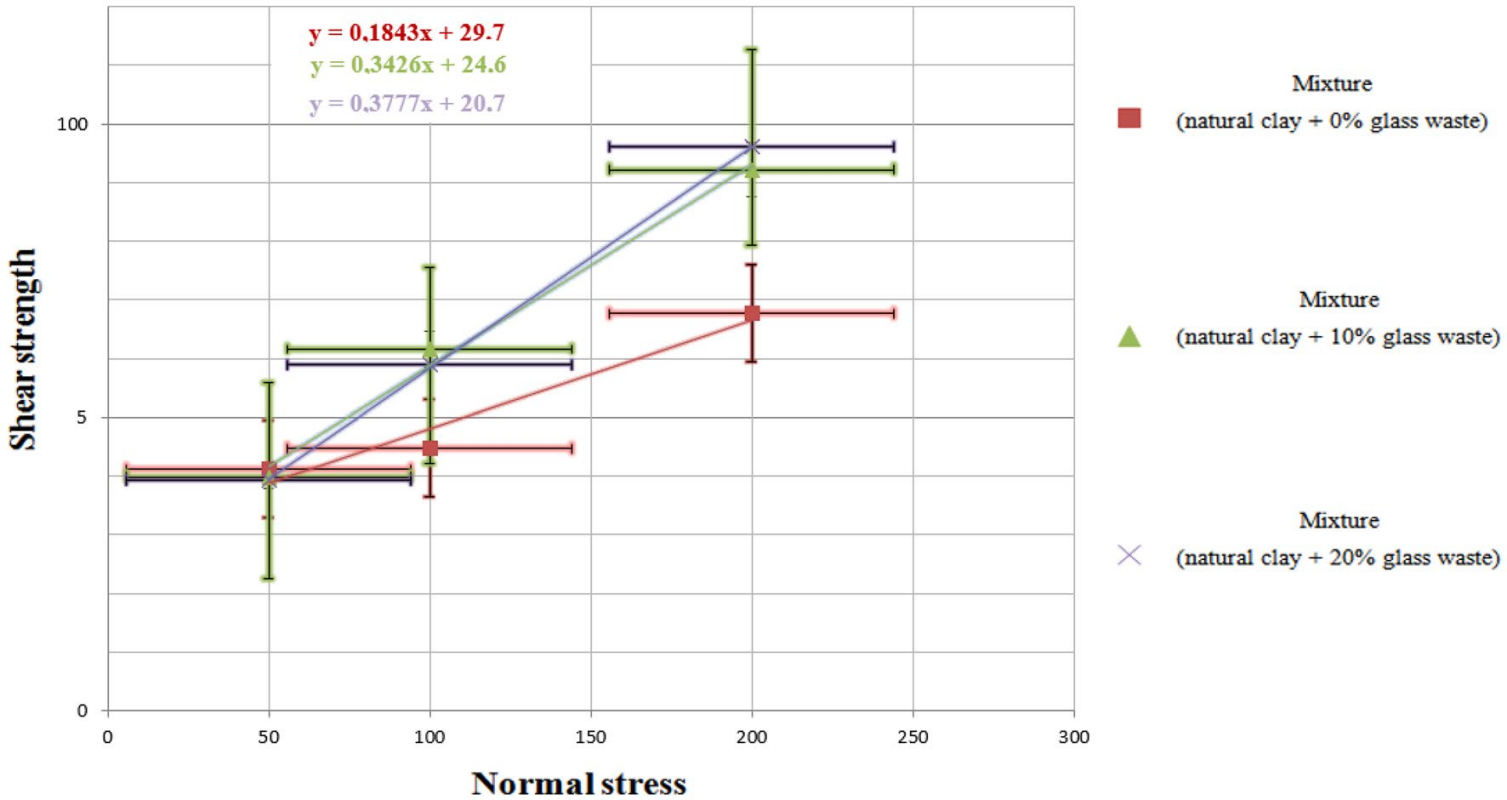
Intrinsic curves of the tested samples and values of c and ϕ.

Direct shear tests using a shear box^55^ are conducted to examine the resistance of the mixtures to tangential forces generated by traffic, especially heavy vehicles. These tests enable the determination of the mechanical shear characteristics, including the study of cohesion (**c**) and the angle of internal friction (**φ**), and the results are depicted in Fig. 13.

The results obtained from the shear tests (Fig. 13) indicate that the natural clays studied (clay) exhibit a maximum angle of friction of 21° with 20% glass. The addition of glass leads to an improvement in the friction angle, reaching a minimum value of 19° with 10% glass. This improvement can be attributed to the irregular shape and high angles of the glass waste used. As a result, the points of contact between the natural clays experience increased friction in Table 7.

**Table 7:** Tested samples and values.

Sample	Cohesion C (kPa)	The angle of friction ϕ (°)	Allowable stress σ_ad(KN/m^2^)
Mixture (clay + 0% glass waste)	30.00 ± 1.0	10.00 ± 0.5	1.19 ± 0.01
Mixture (clay + 10% glass waste)	25.00 ± 0.9	19.00 ± 0.5	1.83 ± 0.01
Mixture (clay + 20% glass waste)	21.00 ± 0.8	21.00 ± 0.5	1.89 ± 0.01

The permissible stress (qα) is determined based on the mechanical characteristics using a general formula provided (1)^56,57^.

1 $$ q_{a} = \frac{1}{{{\text{F}}\left( {\frac{1}{{2 \times {\upgamma } \times {\text{S}}_{{\upgamma }} \times {\text{B}} \times {\text{N}}_{{\upgamma }} + {\upgamma } \times {\text{D}} \times {\text{S}}_{{\text{q}}} \times {\text{N}}_{{\text{q}}} + {\text{C}} \times {\text{S}}_{{\text{c}}} \times {\text{N}}_{{\text{c}}} }}} \right)}} $$

With:

$$ \left\{ {\begin{array}{ll} {{\text{S}}_{{\text{y}}} = {1} - 0.{2} \times {\text{B}}/{\text{L}}} \\ {{\text{S}}_{{\text{q}}} = {1}} \\ {{\text{S}}_{{\text{c}}} = {1} + 0.{2}\;{\text{B}}/{\text{L}}} \\ \end{array} } \right. $$

The incremental introduction of glass debris results in an observable decline in the cohesion of the mixtures. As a result of the discontinuities introduced by the glass into the clay particles, their inherent cohesion is diminished. As a result, the integration of glass materials results in a significant enhancement in the angles of internal friction, accompanied by a marginal decrease in cohesion. The combination of the glass waste and the overall improvement in tensile resistance observed in the composites makes them potentially viable for application as pavement layers. The modification in cohesion occurs as a direct result of the interaction between fragments of glass and clay. As a consequence of these interactions, the cohesive forces within the mélange are disrupted, and cohesion is diminished. Notwithstanding the reduction in cohesion, the substantial improvement in internal friction angles substantially fortifies the mixture's resistance to shear forces.

The improved shear resistance is particularly valuable in the context of road construction, where pavement layers are subjected to continuous traffic-induced stresses. The increased internal friction angles enhance the mixture's resistance to deformations and slippage, contributing to the long-term durability and stability of the pavement layers. The addition of glass waste to clay mixtures induces changes in mechanical properties by enhancing internal friction angles and modifying cohesion. These changes bolster shear resistance, making these mixtures suitable candidates for use as construction materials in pavement layers. However, a thorough assessment of their performance under specific usage conditions is recommended to ensure their suitability for road construction requirements.

## Conclusion

This comprehensive study on the integration of recycled glass waste into expansive clay intended for road construction highlights several key aspects.. The addition of recycled glass waste brings about significant changes in the physical, mechanical, and structural properties of the modified clay. The results of Proctor and CBR tests reveal variations in optimal dry density, water content, and bearing capacity of the clay based on the quantity of added glass waste. X-ray diffraction (XRD) analyses show alterations in the characteristic diffraction peaks of the clay, suggesting complex interactions between the glass waste and clay minerals. These interactions are further corroborated by infrared analysis results, indicating changes in absorption bands that reflect chemical and structural rearrangements within the clay matrix.

However, rigorous proportional optimization and a detailed knowledge of the interactions between glass waste and clay minerals are required to optimize advantages while limiting negatives. Finally, this work emphasizes the need of a multidisciplinary approach and the careful use of several characterisation methodologies when assessing the influence of recycled materials on building material attributes. The results of this study may help guide judgments about the design and deployment of these mixes in road building while supporting a sustainable and ecologically friendly approach.

### Supplementary Information

Supplementary Information.

## Data Availability

All data generated or analyzed during this study are included in this published article.
