# Supplementary material for: Optimization of glass scrap recovery and reuse in road construction for promising physicochemical stabilization
Source: Sci Rep. 2024 Jun 5;14:12925. doi: 10.1038/s41598-024-62862-x (PMC11153621; doi:10.1038/s41598-024-62862-x)
Supplement: Supplementary file 1 — Supplementary Information. [file 41598_2024_62862_MOESM1_ESM.docx]

**Supplementary Material**


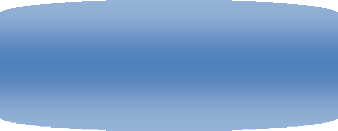

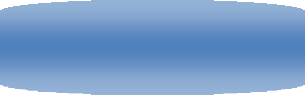

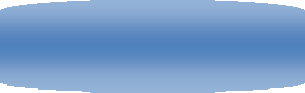

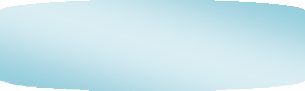

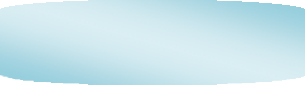

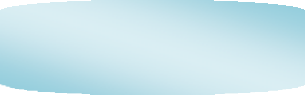

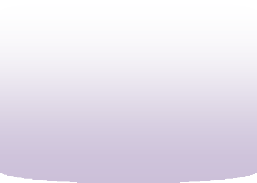

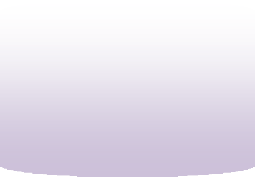

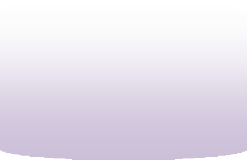

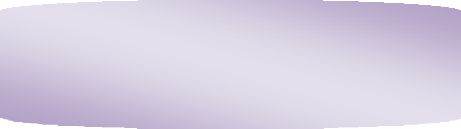

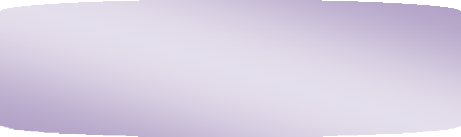


Base materials (clay)

The glass

Types of tests

Mechanical test

Mechanical test

Mechanical test

***Modified Proctor Test***

***CBR test***

**Shear test**

**Mixture (natural clay+ (0%, 10%, 20%) glass waste)**

**Results**

**Fig SI.1.** Flowchart of the study on the treatment of clay with glass.

**
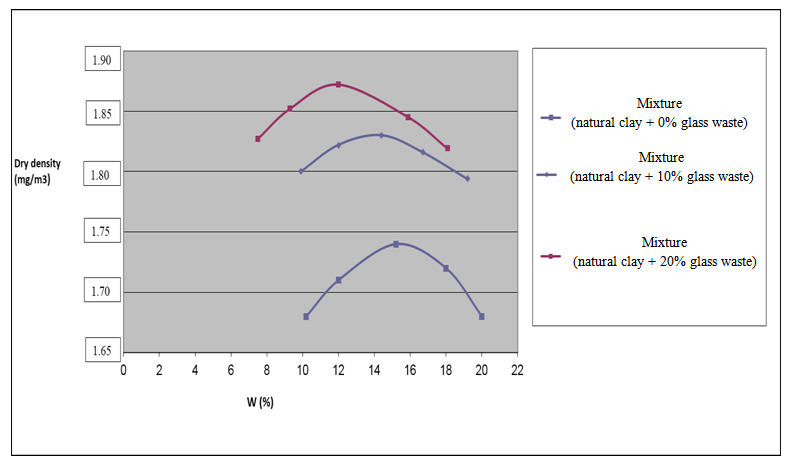
**

**Fig. SI.2** Proctor curves of studied mixtures

**
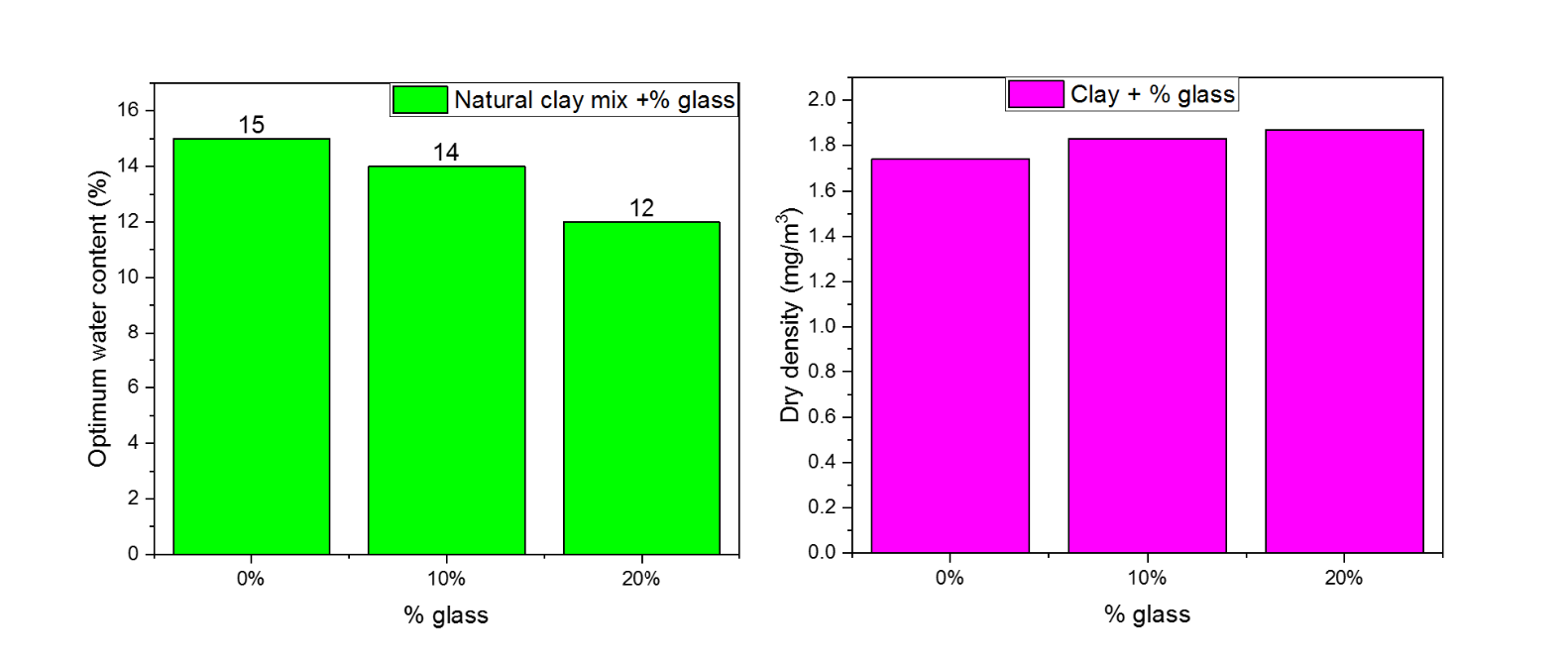
**

**Fig. SI.3** Evolution of compaction parameters as a function of glass content


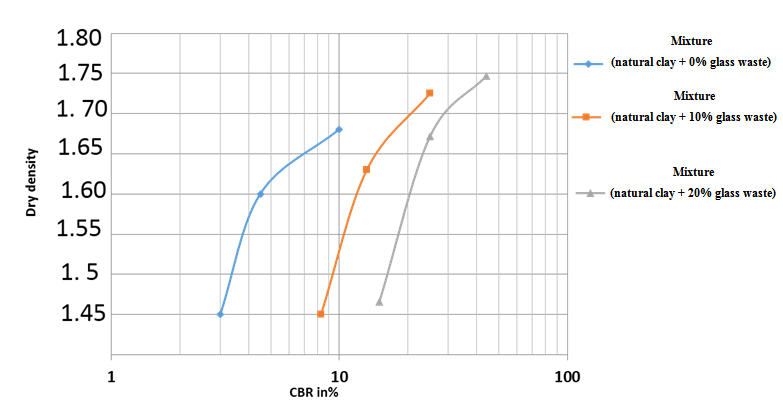


**Fig. SI.4** Influence of mixture addition on CBR curves after immersion
